# Heme oxygenase-1 and its metabolites affect pancreatic tumor growth *in vivo*

**DOI:** 10.1186/1476-4598-8-37

**Published:** 2009-06-09

**Authors:** Philipp Nuhn, Beat M Künzli, René Hennig, Tomas Mitkus, Tadas Ramanauskas, Rainer Nobiling, Stefan C Meuer, Helmut Friess, Pascal O Berberat

**Affiliations:** 1 Department of Surgery, Technische Universität München, Munich, Germany; 2 Department of Surgery, University of Heidelberg, Heidelberg, Germany; 3 Institute of Immunology, University of Heidelberg, Heidelberg, Germany

## Abstract

**Background:**

Pancreatic cancer (PaCa) is a fatal human cancer due to its exceptional resistance to all current anticancer therapies. The cytoprotective enzyme heme oxygenase-1 (HO-1) is significantly overexpressed in PaCa and seems to play an important role in cancer resistance to anticancer treatment. The inhibition of HO-1 sensitized PaCa cells to chemo- and radiotherapy *in vitro*.

Therefore, we investigated the effects of HO-1 and its metabolites biliverdin, carbon monoxide and iron on PaCa cells.

PaCa cell lines with divergent HO-1 expression patterns were used in a murine orthotopic cancer model. HO-1 expression and activity was regulated by zinc (inhibition) and cobalt (induction) protoporphyrin. Furthermore, the influence of cellular HO-1 levels and its metabolites on effects of standard chemotherapy with gemcitabine was tested *in vivo *and *in vitro*.

**Results:**

High HO-1 expression in PaCa cell lines was associated with increased chemoresistance *in vitro*. Chemoresistance to gemcitabine was increased during HO-1 induction in PaCa cells expressing low levels of HO-1. The inhibition of HO-1 activity in pancreatic tumors with high HO-1 boosted chemotherapeutic effects *in vivo *significantly. Furthermore, biliverdin and iron promoted PaCa resistance to chemotherapy. Consequently, specific iron chelation by desferrioxamine revealed profound anticancerous effects.

**Conclusion:**

In summary, the inhibition of HO-1 and the chelation of iron in PaCa cells were associated with increased sensitivity and susceptibility of pancreatic tumors to chemotherapy *in vivo*. The metabolites biliverdin and iron seem to be involved in HO-1-mediated resistance to anticancer treatment. Therefore, HO-1 inhibition or direct interference with its metabolites may evolve new PaCa treatment strategies.

## Background

Pancreatic cancer is one of the most serious human cancers with an incidence rate that almost equals its mortality rate [1]. Most patients show advanced unresectable tumors at the time of diagnosis. Despite efforts in the last decades, surgical resection of the tumor is still the only approach for potential cure, as PaCa is nearly insensitive to standard radiation and chemotherapeutic treatments [2].

Heme oxygenase-1 (HO-1) is the inducible isoform of the three heme oxygenases (HO-1, HO-2 and HO-3) that catalyze the degradation of heme to biliverdin, carbon monoxide (CO) and free iron [3]. Subsequently, the cytosolic enzyme biliverdin reductase converts biliverdin into bilirubin. Free iron is promptly sequestered by ferritin [4,5].

HO-1 and its products possess anti-inflammatory and anti-apoptotic properties [6-8]. Furthermore, HO-1 regulates cell proliferation [9] and functions as an important proangiogenic mediator [10-12].

Elevated HO-1 expression and activity were observed in various tumor tissues such as human renal cell carcinoma [13], prostate tumors [14], lymphosarcomas [15] and PaCa [16]. In response to radio- and chemotherapy, HO-1 expression is potently increased [16,17]. Therefore, it was hypothesized that HO-1 and its products may have important roles in tumor progression and formation of metastases as well as resistance to anticancer therapy. It was shown that the antioxidative and antiapoptotic properties of HO-1 are related to these effects [12,18]. In an experimental murine PaCa model, HO-1 accelerated tumor growth by increasing angiogenesis [10]. Furthermore, human PaCa tissues showed increased expression of HO-1 in tumor cells and in tumor associated immunocytes. Targeted knockdown of HO-1 gene expression *in vitro *was associated with growth inhibition of PaCa cells and increased chemo- and radiosensitivity of tumor cells significantly [16].

In this study we tested potential anticancerous effects of HO-1-inhibition *in vivo *and therefore aimed for a potential therapeutic approach for sensitizing tumors to chemotherapy. Furthermore, the role of HO-1 metabolites in this context should be analyzed.

## Results

### Pancreatic cancer cell lines PANC-1 and S2-013 show divergent HO-1 expression levels

**Figure 1:**
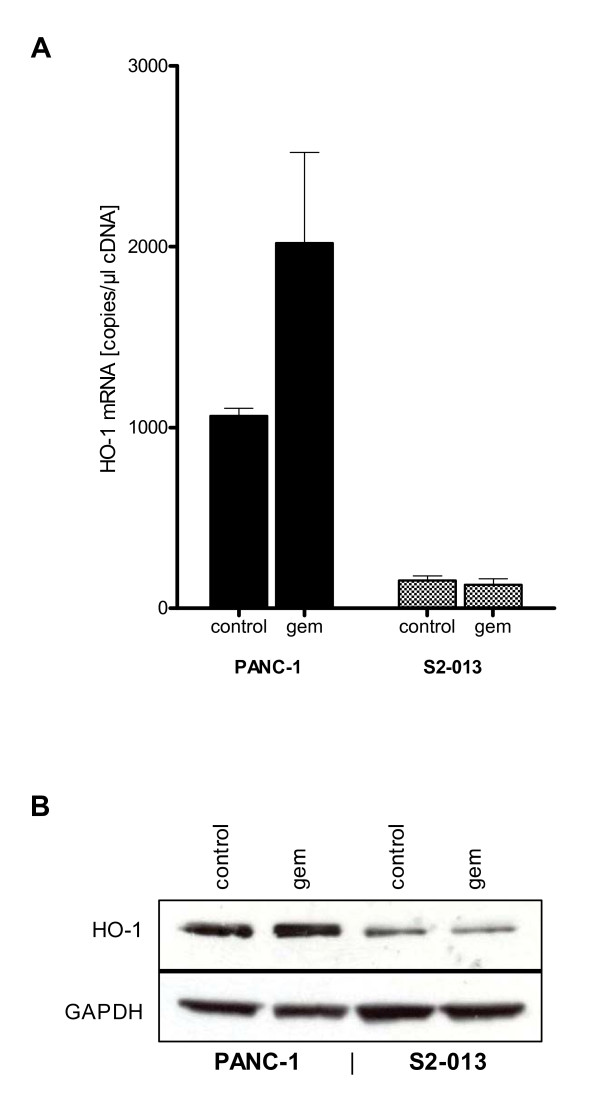
**Pancreatic cancer cell lines PANC-1 and S2-013 show divergent HO-1 expression levels**. mRNA extractions from cells for QRT-PCR and cell lysates for Western blot were prepared from untreated cells and cells treated with gemcitabine. (1A) No treatment of PANC-1 cells displayed high native mRNA expression of HO-1, whereas HO-1 expression was only at levels of detection in S2-013. Treatment with gemcitabine increased mRNA expression of HO-1 in PANC-1 cells but did not alter the expression level in S2-013 cancer cells. (1B) Protein analyses in resting condition revealed high native expression of HO-1 in PANC-1 but faint expression in S2-013. Under gemcitabine conditions protein expression of HO-1 was slightly induced in PANC-1 cell line but did not alter HO-1 protein expression in S2-013 cell line.

To confirm expression of HO-1 in the present cell lines and to check the effect of gemcitabine treatment, mRNA- and protein levels were examined using RT-PCR and Western blot analysis. Cell lines showed divergent mRNA-expression levels of HO-1 (Fig. 1A). Native expression was high in PANC-1 (1063.33 ± 43.10 copies/μl cDNA), whereas S2-013 (153.67 ± 24.54 copies/μl cDNA) indicated low levels of HO-1 expression. HO-1 mRNA expression was induced in PANC-1 cancer cells treated with gemcitabine.

Protein analyses revealed high native expression of HO-1 in PANC-1 in comparison to S2-013 cell line, which showed only faint expression. Under gemcitabine conditions protein expression of HO-1 was slightly induced in PANC-1 cell line but did not alter in S2-013 cell line (Fig. 1B).

### Different HO-1 expression levels are associated with variable susceptibility to chemotherapy

**Figure 2:**
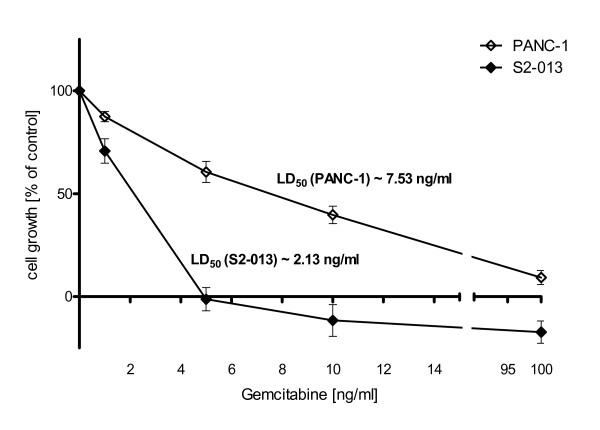
**Different HO-1 expression levels are associated with variable susceptibility to chemotherapy**. Cell viability was assessed 72 h after application of gemcitabine via MTT assay. Both cell lines showed dose dependent growth inhibition under treatment with gemcitabine. S2-013 cell line with low native HO-1 expression was significantly more susceptible to gemcitabine (LD50 [S2-013] ~2.13 ng/ml) than PANC-1 cell line with high native HO-1 expression (LD50 [PANC-1] ~7.53 ng/ml).

Both cell lines revealed profound growth inhibition under treatment with gemcitabine (Fig. 2). PANC-1 cell line (LD_50 _[PANC-1] ~7.53 ng/ml) with high native HO-1 expression showed significantly greater chemoresistance than S2-013 cell line (LD_50 _[S2-013] ~2.13 ng/ml) with low native HO-1 expression.

### Inhibition of HO-1 activity leads to increased susceptibility to chemotherapy *in vitro*

To prove cytoprotective properties of HO-1 to anticancer treatment, the HO-1 inductor CoPP and ZnPP, which has been shown to inhibit HO-1 activity *in vitro *and *in vivo *[7,19-21], were added to tumor cells 24 h prior to the application of gemcitabine.

**Figure 3:**
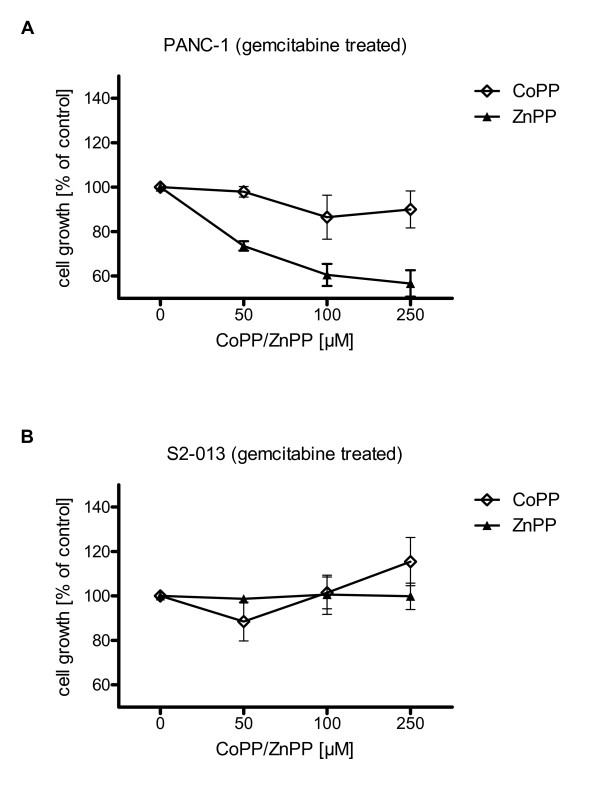
**Inhibition of HO-1 activity leads to increased susceptibility to chemotherapy *in vitro***. Cell viability was assessed 72 h after application of gemcitabine via MTT assay. (3A) Under gemcitabine treatment (LD50 dose) application of ZnPP revealed a dose dependent growth inhibition of PANC-1 cancer cells, whereas application of CoPP had no significant effect on cell proliferation. (3B) In S2-013 cell line implementation of CoPP led to increased cell proliferation, whereas ZnPP had no significant effect.

In the PANC-1 cell line, application of ZnPP under gemcitabine treatment revealed a dose dependent growth inhibition (-43.30 ± 5.89%), whereas application of CoPP had no significant effect on cell proliferation (-9.99 ± 8.31%) (Fig. 3A). In the S2-013 cell line implementation of CoPP increased cell proliferation (+15.49 ± 10.88%), whereas ZnPP had no significant effect (+0.11 ± 5.93%) (Fig. 3B).

### Inhibition of HO-1 activity leads to increased susceptibility to chemotherapy of solid tumor growth

The cell lines S2-013 and PANC-1 were utilized for orthotopic tumor cell injection in nude mice, as previously described for S2-013 cells [22].

**Figure 4:**
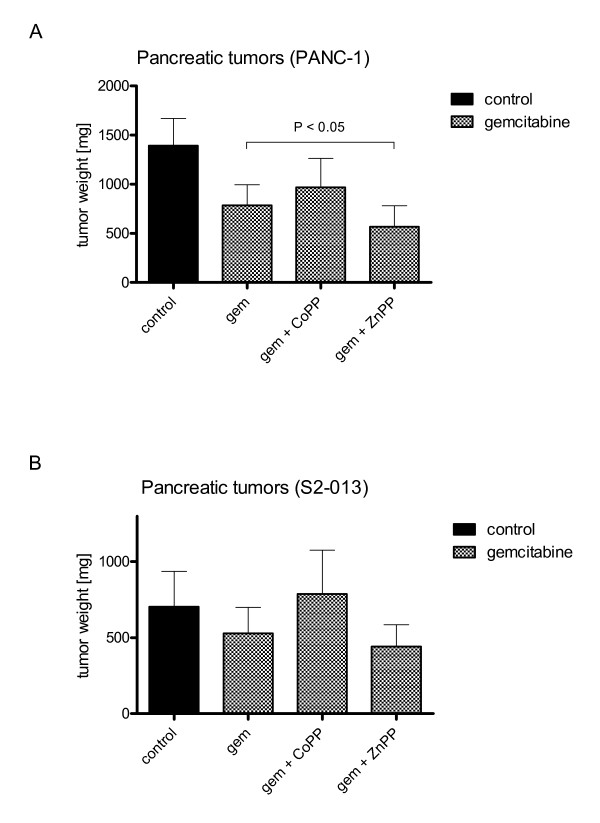
**Inhibition of HO-1 activity leads to increased susceptibility to chemotherapy of solid tumor growth**. Primary pancreatic tumor weights were assessed 4 and 8 weeks after orthotopic tumor implantation of cancer cells in nude mice (n = 60, 15 per group). (4A) ZnPP treatment reduced tumor growth in gemcitabine treated mice after PANC-1 cancer cell implantation significantly, compared to gemcitabine treated tumors only. (4B) Administration of gemcitabine in combination with CoPP or ZnPP in mice implanted S2-013 cancer cells did not significantly change tumor weights in comparison to gemcitabine treatment alone. The use of ZnPP in combination with gemcitabine showed a trend towards lower tumor growth.

Mice receiving PANC-1 cancer cells treated with gemcitabine showed significantly reduced tumor growth in comparison to untreated control (-43.61 ± 15.12%) (Fig. 4A). Inhibition of HO-1 activity by ZnPP boosted anticancerous effects of gemcitabine resulting in significant reduction of tumor growth in gemcitabine treated mice with PANC-1 tumors (-59.26 ± 15.32%) (P < 0.05) as opposed to gemcitabine treated tumors only (-43.61 ± 15.12%). Administration of CoPP in combination with gemcitabine tended to increased tumor weights in comparison to gemcitbine treatment only.

In S2-013 cell line, administration of CoPP in combination with gemcitabine facilitated pancreatic tumor progression tending to result in higher tumor weights in comparison to gemcitabine alone. The use of ZnPP in combination with gemcitabine tended to result in lower tumor growth (Fig. 4B) but was not significantly different.

### HO-1 metabolites make pancreatic cancer cells more resistant to chemotherapy

To study possible effects of HO-1 metabolites biliverdin, CO and free iron on growth behavior of PaCa cell lines further proliferation experiments were performed.

**Figure 5:**
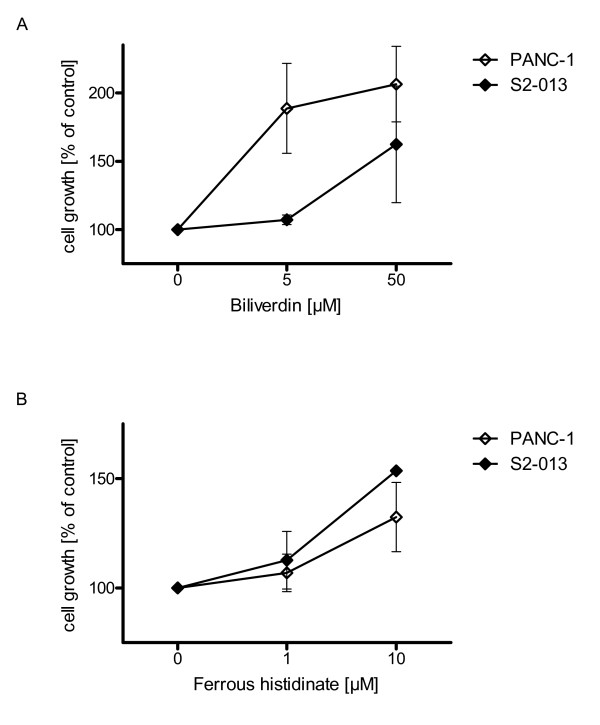
**HO-1 metabolites mediate resistance effects to chemotherapy**. Cell growth was assessed 72 h after application of gemcitabine via MTT assay. (5A) Biliverdin administration biliverdin [5 and 50 μmol/l] resulted in increased proliferation of cancer cell line PANC-1 and S2-013, and therefore reduced the chemotherapeutic effect of gemcitabine (LD50 dose). (5B) Administration of ferrous histidinate revealed increased cell proliferation of PANC-1 and S2-013 cancer cells.

Application of biliverdin [5 and 50 μmol/l] resulted in increased cell proliferation for PANC-1 (+106.43 ± 27.68%) and S2-013 (+62.45 ± 42.67%) cancer cells (Fig. 5A). *In vivo*, implementation of biliverdin tended to increase tumor growth (S2-013) in gemcitabine treated mice (data not shown).

Ferrous histidinate was used to study the effects of the HO-1 metabolite iron. Administration of ferrous histidinate in high concentrations increased the proliferation of PANC-1(+32.45 ± 15.80%) and S2-013 (+53.63 ± 0.77%) cancer cells (Fig. 5B). CO exposure to PANC-1 and S2-013 showed a nonsignificant tendency towards increased cancer cell growth (data not shown).

### Depletion of iron increases susceptibility to chemotherapy *in vivo *and *in vitro*

**Figure 6:**
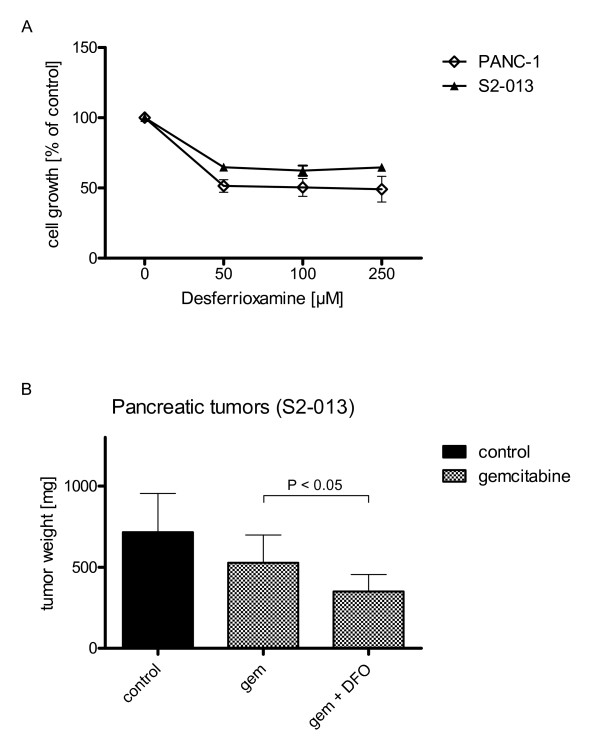
**Depletion of iron increases susceptibility to chemotherapy *in vivo *and *in vitro***. Cell growth was assessed 72 h after application of gemcitabine via MTT assay. (6A) Implementation of DFO resulted in dose dependent profound growth inhibition of both cell lines. (6B) Primary pancreatic tumor weights were assessed 4 weeks after orthotopic tumor implantation of S2-013 cancer cells in nude mice (n = 60; 15 mice per group). DFO plus gemcitabine reduced the tumor growth significantly (tested for S2-013 cell line only).

The use of DFO resulted in significantly reduced cell proliferation in PANC-1 (-50.91 ± 15.93%) and S2-013 (-35.26 ± 4.64%) cell lines *in vitro *(Fig. 6A). Furthermore, administration of DFO was associated with a significantly lower tumor growth *in vivo *in mice implanted S2-013 cell line when combined with gemcitabine (-51.13 ± 14.66%) (Fig. 6B).

## Discussion

The HO-1 system has important cellular functions in self-defense processes resisting a wide range of external stress stimuli and in growth regulation [9,23]. In experimental and clinical approaches for various HO-1 associated disorders [24,25], HO-1 and its metabolites with their anti-inflammatory and anti-apoptotic properties provide protection for cells, tissues and even whole organs. Furthermore, HO-1 inducers influence certain diseases in particular [9]. In transgenic mice, cardiac-specific expression of HO-1 offered protection against ischemia and reperfusion injury [26]. Exogenous administration of HO-1 by gene transfer provided protection against hyperoxia induced lung injury in rats [27].

HO-1 is highly upregulated in different human cancers including pancreatic carcinoma [5,13,14,16]. It has been shown that inhibition of HO-1 expression and activity by siRNA transfection in different PaCa cell lines diminished cell proliferation and enhanced their response to HO-1-inducing radiation and chemotherapeutics [16]. In an experimental mouse model, HO-1 accelerated angiogenesis in human PaCa [10]. HO-1 expression in cancer cells surrounding immunocytes [16] could be associated with promoted local tumor growth as expression of HO-1 in macrophages is correlated with neovascularization [28,29].

Thus, overexpression of HO-1 in PaCa may develop tumor-promoting activity by enhancing cell proliferation, improving resistance to oxidative stress and apoptotic stimuli and increasing angiogenic potential of tumor cells.

The present study demonstrated that high expression levels of native HO-1 were associated with impaired susceptibility to chemotherapy in PaCa cell lines. Furthermore, we confirmed the antiproliferative and sensitizing effect of HO-1 inhibition on PaCa cells *in vitro*. Treatment with HO-1 activity inhibitor ZnPP, in addition to gemcitabine, reduced cell proliferation in PANC-1 expressing high levels of native HO-1.

Moreover, we show that the inhibition of HO-1 activity *in vivo *significantly increased the susceptibility to chemotherapy of solid tumor growth. Treatment with gemcitabine in combination with ZnPP resulted in significantly reduced tumor weights in tumors with high native HO-1 (PANC-1) in comparison to the only gemcitabine treated control group. Inhibition of heme oxygenase-1 activity by zinc protoporphyrin IX reduced tumor growth of LL/2 lung cancer in C57BL mice [30]. ZnPP probably interrupts the direct proliferative effects of HO-1 on the cell cycle [16]. In cultured cells (HA-1), ZnPP incubation increased apoptosis by enhancing expression of p53 protein by the Egr-1 protein, an antiproliferative signaling molecule in tumor cells transactivating the promoter for the p53 gene [31,32]. Typically, antioxidative enzymes like glutathione peroxidase, catalase or superoxide dismutase are downregulated in tumor cells [33-35]. Thus, the HO-1 system, whose activity is inhibited by ZnPP [7,19-21], may play a major role in the defense against oxidative stress generated by chemotherapeutic agents in these cells. In contrast to these findings, induction of HO-1 tended to increase tumor growth *in vivo *and *in vitro *in tumors with low native HO-1 expression and impaired susceptibility to chemotherapy.

These *in vivo *results support the suggested potential therapeutic role of HO-1 inhibition in PaCa. Therefore, we investigated whether anticancerous effects of HO-1 were mediated by the heme degradation products bilirubin, CO and iron.

Biliverdin showed protective effects against gemcitabine on cell proliferation in all cell lines. *In vivo *(S2-013) the administration of bilirubin in combination with gemcitabine tended to increase tumor growth (data not shown). Bilirubin is a potent antioxidant that scavenges free radicals [36-38]. The PaCa cells seem to utilize this antioxidant produced by HO-1 to circumvent oxidative stress generated by the chemotherapeutic agent.

Carbon monoxide (CO) did not show influence on cell proliferation during gemcitabine treatment *in vitro*. CO, a signal molecule triggering a series of signal transductions, has nonetheless been shown to provide protection of cells against injury of various kinds [9] both *in vitro *and *in vivo *due to its antiapoptotic and anti-inflammatory activities. CO inhibited TNFα-induced apoptosis in cultured fibroblasts [39], provided protection against hyperoxic lung injury in rats [40] and suppressed the rejection of mouse-to-rat cardiac transplants [41]. Inhibition of HO-1 reversed the inhibitory effect of IL-10 on TNFα production[42] Therefore, inhibition of HO-1 in tumor tissues may increase TNFα production, hence increasing the inflammatory response of the host. In our experiments CO seems not to play a central role in PaCa growth. However, the observed tendency of increased proliferation under CO treatment could justify further investigations.

Administration of ferrous histidinate had proliferative effects *in vitro*. This is not surprising as iron containing proteins catalyze key reactions involving energy metabolism, respiration and DNA synthesis [43]. Iron is essential for cellular growth and division, especially for rapidly proliferating cancer cells. We showed that iron chelators can target this altered iron metabolism of cancer cells [43]. In the present study DFO had significant and profound antiproliferative effects on tumor cell growth in all cell lines. In combination with gemcitabine DFO boosted significantly the anti-proliferative effects of gemcitabine in tumor cells. Also, tumors from S2-013 cancer cells treated with a combination of gemcitabine with DFO had significantly decreased tumor weights compared to gemcitabine treated mice. DFO already showed anti-tumoral effects on other tumors *in vitro *as well as in limited clinical trials [44]. DFO showed antileukemic effects on human myeloid leukemia cell lines [45]. It also led to growth inhibition in neuroblastoma cells [46] and showed anitumor activity in neuroblastoma patients [47].

## Conclusion

Our results showed that the inhibition of HO-1 in tumors related to reduced tumor growth and increased responsiveness of PaCa to chemotherapy *in vivo*. Our study further demonstrated that the anti-inflammatory and antiapoptotic activities of HO-1, providing resistance of PaCa cells to cellular stress from host immunity or anticancer therapies, were at least partially exerted by its metabolite biliverdin. Furthermore, the production of iron seems to promote tumor growth. Based on this finding, implementation of iron chelators displayed significant antitumoral effects. Depletion of iron increased susceptibility to chemotherapy *in vivo *and *in vitro*. Therefore, HO-1 inhibition or direct interference with its metabolites may evolve as new tactics in anticancer treatment of PaCa.

## Methods

### Reagents

Gemcitabine (Lilly, Fegersheim, France) was dissolved in PBS and diluted in 0.9% NaCl to a stock concentration of 24 mg/ml and stored at 4°C until use. Cobalt protoporphyrin (CoPP), zinc protoporphyrin (ZnPP) and biliverdin hydrochloride (Frontier Scientific Inc., Logan, UT, USA) were dissolved in 100 mM NaOH, subsequently adjusted to a pH of 7.4 with 1 M HCl, and diluted in 0.9% NaCl. The stock solutions of CoPP, ZnPP (1 mg/mL) and biliverdin (10 mM) were aliquoted and stored at -80°C until used. Desferrioxamine (DFO) mesylate (Sigma Aldrich GmbH, Deisenhofen, Germany) was dissolved in aqua ad injectabilia to a stock concentration of 0.5 g/mL and diluted with 0.9% NaCl. All reagents were protected from light exposure. Fe(II)-histidinate solution was freshly prepared in PBS containing FeSO_4 _(10 mM) and L-histidine (100 mM) (Sigma Aldrich GmbH, Deisenhofen, Germany).

### Cell culture

The common human PaCa cell lines PANC-1 with ductal cell origin and S2-013, derived from a metastatic liver tumor of human pancreatic carcinoma, were cultured using standard cell culture techniques, conditions and media, supplemented with 10% fetal calf serum albumin (PAN Biotech GmbH, Aidenbach, Germany), 100 U/mL penicillin and 100 μg/mL streptomycin (Invitrogen, Karlsruhe, Germany), as previously described [48].

PANC-1 were obtained from American Type Cell Collection (Manassas, Virginia, USA), S2-013 cells were provided by Dr. M.A. Hollingsworth (Eppley Cancer Institute, Omaha, NE, USA). Cells were incubated for at least 72 hours before each experiment and harvested with trypsin/EDTA (0.05% Trypsin, 53 mM EDTA) (Invitrogen, Karlsruhe, Germany). S2-013 tumor cells were harvested at 90% confluency as previously described [22].

### Real-time Light Cyclers QRT-PCR

All reagents and equipment for mRNA/cDNA preparation were purchased from Roche Applied Science Diagnostics (Mannheim, Germany). mRNA extractions from cells were prepared by automated isolation using the MagNA Pure^® ^LC instrument and isolation kits I, cDNA was prepared using the first strand cDNA synthesis kit according to the manufacturer's instructions. Real-time PCR was performed with the LightCycler^® ^FastStart DNA SYBR Green kit. All primers were obtained from Search-LC (Heidelberg, Germany). The calculated number of specific transcripts was normalized to the housekeeping genes cyclophilin B and hypoxanthine guanine phosphoribosyltransferase, and expressed as number of copies per ml of input cDNA.

### Immunoblotting

Cell lysates were prepared and sodium dodecyl sulfide-polyacrylamide gel electrophoresis techniques were used to separate proteins exactly as previously described [49]. The membranes were probed with primary anti-HO-1 mouse monoclonal antibody (BD Biosciences Transduction Laboratories, Lexington, KY, USA). Secondary peroxidase-conjugated affinity purified goat anti-mouse antibody (Jackson ImmunoResearch Laboratories, Inc., West Grove, Pennsylvania, USA) was used for detection, as previously described [49]. Equal gel loading was confirmed by analyses with glyceraldehyde-3-phosphate dehydrogenase antibody (Santa Cruz Biotechnology, Inc., Santa Cruz, California).

### *In vivo *studies

Six- to eight-week-old female athymic nude (NU/NU) mice (n = 135), each weighing at least 20 g, were purchased from Charles River Laboratories, Inc. (Wilmington, Massachusetts, USA). Mice were utilized in accordance with the principles laid down in the European Community's Council Directives and approved by the local administration (reference 35-9185.81/G-138/04). Animals were controlled on a daily basis and checked for distress symptoms, as previously described [49].

#### Orthotopic Tumor Implantation

Mice were narcotized intraperitoneally with 0.05 ml of a mixture of 0.4 ml of S-ketamine hydrochloride (Ketanest S^®^, 25 mg/ml; Pfizer, Karlsruhe, Germany), 0.1 ml of xylazine hydrochloride (Rompun^® ^2%; Bayer HealthCare, Leverkusen, Germany), and 0.5 ml of NaCl. Orthotopic tumor cell implantation was performed as described elsewhere [22]. In brief, under sterile conditions, mice were fixed in supine position and the abdomen was disinfected with alcohol swabs. Following a 0.5 cm midline incision and exposure of the situs the stomach was retracted to expose the pancreas. Tumor cells (5 × 10^5 ^cells in 10 μl of DMEM) were injected into the duodenal lobe using a monoject 200 27-gauge × 1/2 in. polypropylene hub hypodermic needle (Kendall, Mansfield, Massachusetts, USA) and a 50 μl gastight glass syringe (Hamilton Company, Reno, Nevada, USA). The successful injection was confirmed under a stereo microscope. After gentle reposition of the stomach into the peritoneal cavity, the incision was sutured in two layers with vicryl-coated rapide sutures 4-0 (Ethicon, Inc., Somerville, New Jersey, USA). Mice were closely monitored until awakened from narcosis and afterwards transferred back to standardized animal care.

#### Treatment

One day after surgical orthotopic tumor cell implantation, mice were randomized into the indicated groups. In both cell lines, S2-013 and PANC-1, there were at least four groups: control (group I), gemcitabine (group II), gemcitabine in combination with ZnPP (group II) and gemcitabine in combination with CoPP (group IV) (Table 1, Table 2).

**Table 1:** Different categories of treatment for PANC-1 cell line

**#**	**Group**	**n**	**Treatment**
**I**	control	15	-
**II**	gemcitabine*	15	gemcitabine
**III**	gemcitabine/CoPP^†^	15	gemcitabine/CoPP
**IV**	gemcitabine/ZnPP^†^	15	gemcitabine/ZnPP

* Gemcitabine (5 doses, 60 mg/kg per dose) on days 11, 14, 17, 20 and 23.

^† ^CoPP or ZnPP (5 mg/kg per dose) every alternative day from day 9 to day 23.

**Table 2:** Different categories of treatment for S2-013 cell line

**#**	**Group**	**n**	**Treatment**
**I**	control	15	-
**II**	gemcitabine*	15	gemcitabine
**III**	gemcitabine/CoPP^†^	15	gemcitabine/CoPP
**V**	gemcitabine/DFO^‡^	15	gemcitabine/DFO
**IV**	gemcitabine/ZnPP^†^	15	gemcitabine/ZnPP

* Gemcitabine (4 doses, 60 mg/kg per dose) on days 7, 10, 13 and 17.

^† ^CoPP, ZnPP (5 mg/kg per dose) or biliverdin (50 μmol/kg per dose) every alternative day from day 5 to day 17.

^‡ ^DFO administration via an intraperitoneal osmotic pump (250 mg/kg per day for two weeks) from day 7 onwards.

In both cell lines, group I (control) received intraperitoneal injections of the vehicle only. Group II (gemcitabine) received intraperitoneal injections of gemcitabine (60 mg/kg per dose) on days 7, 10, 13, and 17 (S2-013, total of 4 doses) and on days 11, 14, 17, 20 and 23 (PANC-1, total of 5 doses) after tumor injection. Group III (gemcitabine with ZnPP) was additionally administered ZnPP (5 mg/kg per dose) every alternative day starting two days prior to the first dose and finishing after last dose of gemcitabine. Group IV (gemcitabine with CoPP) received injections of CoPP (5 mg/kg per dose) in the same regimen as group III (Table 1, Table 2).

For the S2-013 cell line treatment combining gemcitabine with desferrioxamine (DFO) (group V) was tested: Mice of group IV (gemcitabine with DFO) were implanted osmotic pumps (Alzet^© ^Osmotic Pump 1002; Alzet Osmotic Pumps, Cupertino, California, USA) for DFO delivery (250 mg/kg per day for two weeks) subcutaneously on day 5 after tumor implantation (not tested for PANC-1 cell line).

#### Technical read-out of pancreatic tumors

Fifteen mice were assigned to each group and treated for 4 weeks in case of S2-013 and 8 weeks in case of PANC-1 cancer cell treatment, at which time the experiment was terminated. Mice were euthanized and staging and imaging of implanted and newly formed tumor masses were performed as previously described [22]. Peritoneal and pancreatic tumors were weighed, measured and tumor volume (data not shown) was calculated as previously described [22]. Tissues were fixed in 4% formaldehyde and then transferred to 70% ethanol. Afterwards tissues were paraffin-embedded according to histology standard protocols.

### Cell proliferation assay

The 3-(4,5-Dimethylthiazol-2-yl)-2,5-diphenyltetrazolium bromide (MTT) (Sigma Aldrich, Deisendorf, Germany) cell proliferation assay was done to assess cell proliferation and to evaluate the effect of HO-1 and its metabolites on cell proliferation rate. Cells were seeded in 96-well culture plates at densities of 3,000 cells per well for S2-013 and 4,000 cells per well for PANC-1. Following incubation overnight, metabolites and gemcitabine were added at indicated concentrations and incubated for 72 h, before 20 μl MTT (5 mg/ml in PBS; pH 7.4) were added to each well for 4 h. Formazan products were solubilized with acidic isopropanol, and the optical density was measured at 570 nm. The absorbance was corrected for blank readings. All experiments were carried out in sextuplicate and were repeated at least three times.

### Statistical analysis

The data are presented as mean ± standard deviation (SD). P < 0.05 was considered statistically significant. The Mann-Whitney test was employed to compare differences between two independent groups. Multiple comparisons of tumor weights were tested using Kruskal-Wallis ANOVA on ranks as a nonparametric test. Dunn's post test was used as a post-hoc test. Statistical analysis of data was performed and graphs were created using the GraphPad Prism Software 5.01 (GraphPad Software, Inc., San Diego, CA, USA).

## Competing interests

The authors declare that they have no competing interests.

## Authors' contributions

PN performed the *in vitro *and *in vivo *studies and drafted the manuscript. BMK participated in drafting the manuscript and coordination of the study. RH and RN helped to establish the *in vivo *model. TM and TR participated in performing the *in vivo *studies. SCM and HF participated in coordination of the study. POB designed the study and coordinated and helped to draft the paper. All authors read and approved the final manuscript.
